# Temperature-Resolved Proton Transfer Reactions of Biomolecular Ions

**DOI:** 10.5702/massspectrometry.A0083

**Published:** 2020-03-31

**Authors:** Shinji Nonose

**Affiliations:** 1 Graduate School of Nanobioscience, Yokohama City University, 22–2 Seto, Kanazawa-ku, Yokohama 236–0027, Japan

**Keywords:** peptide ions, protein ions, proton transfer reaction, temperature dependence

## Abstract

Temperature-resolved proton transfer reactions of multiply-protonated angiotensin I, disulfide-intact and -reduced lysozyme, and ubiquitin ions to primary, secondary and aromatic amines were examined in the gas phase. Absolute reaction rate constants for the proton transfer were determined from the intensities of the parent and product ions in mass spectra. Dramatic changes were observed in the distribution of product ions and the reaction rate constants. In particular, the rate constants for disulfide-intact lysozyme ions changed more drastically with the change in charge state and temperature compared to the corresponding values for disulfide-reduced ions. Proton transfer reactions were enhanced or suppressed as the result of the formation of complexes between the ions with gaseous molecules, which is related to changes in their conformation with changing.

## INTRODUCTION

The advantage of gas-phase studies is that such systems can be tested using simple model systems. Substantial progress has been made in the past 40 years toward achieving an understanding of simple reactions of small molecular systems through experimental developments.^1,2)^ In particular, proton transfer reactions between a small molecule and a small molecular ion represent typical elementary processes that occur in ion–molecule reactions. The development of electrospray ionization (ESI) mass spectrometry has made it possible to study large biomolecules in the gas phase without any destruction.^3,4)^ Access to biomolecules in the gas phase provides an ideal opportunity to study them in an isolated state. Because protein folding is such a complex process that the connection between the sequence and three-dimensional structure of a protein, and the mechanism of the folding process itself, are not clearly understood despite many years of study. Levinthal has proposed a thought experiment, constituting a self-reference regarding protein folding.^5)^ If a protein were to attain its correctly folded configuration by sequentially sampling all possible conformations, it would require a time longer than the age of the universe to arrive at its correct native conformation, while proteins fold spontaneously on a time scale of less than a millisecond. Anfinsen *et al.* have examined the kinetics of the unfolding and refolding of ribonuclease and has concluded that its native structure is determined only by the amino acid sequence of the molecule, and that the native structure is a unique, in that it is stable and has a kinetically accessible minimum free energy.^6)^ Wolynes has hypothesized that spontaneous protein folding would proceed along a specific funnel on the free energy landscape.^7)^

The native configurations of proteins are influenced by water molecules and the other biomolecules that surround proteins in living systems. Moreover, interactions with surrounding molecules make it more difficult to understand protein folding. By introducing a biological molecule into the gas phase, it is possible to separate its interactions and intramolecular interactions with external molecules and allow them to be examined independently, even though the gas phase is an unusual environment for investigating biological molecular systems. The structures and reactions of gas-phase biomolecules have been studied extensively using various mass spectrometric methods.^8–17)^ Protein conformations and dynamics have been studied extensively by mass spectrometry-based approaches to characterize kinetic intermediates in refolding experiments. A new experimental approach would be expected to allow exact biomolecular structures to be determined in the gas phase.^18)^

Angiotensin I, a relatively small peptide, is composed of 10 amino acid residues, four of which are basic residues. The peptide is known to act as a precursor to angiotensin II in biological systems for blood pressure regulation. Understanding the structures of such molecules, when bound to their receptors is important for determining the ligand–receptor interactions that are involved in receptor activation. Their native states have been qualitatively characterized by NMR.^19,20)^ Numerous gas-phase mass-spectrometric studies of angiotensin I or II ions have been reported.^21,22)^ Lysozyme is a relatively small protein that conains four intramolecular disulfide bonds between cysteine residues. This protein has become one of the most popular and important model systems for understanding the complex nature of protein structure and function. Lysozyme and its derivative of disulfide linkages in solution phase has been investigated by a wide range of complementary biophysical techniques.^23–25)^ The role of disulfide bridges in the formation and maintenance of the three-dimensional folding of lysozyme has been studied. Mass spectrometric studies have shown that conformations in the gas phase are highly dependent on the presence of disulfide bonds.^26–29)^ The conformations of disulfide-intact protein ions are typically highly folded, whereas disulfide-reduced protein ions favor highly diffuse, unfolded forms, since there are no disulfide bonds to impose structural restrictions. Ubiquitin, a small protein, is composed of 76 residues and contains 13 basic residues. The function of ubiquitin is to tag target proteins *via* covalent attachment for proteasomal degradation. Ion mobility mass spectrometric studies have provided evidence to show that the native state ubiquitin conformations are preserved in the gas phase.^10)^

Proton transfer reactions of angiotensin I, disulfide-intact and -reduced lysozyme, and ubiquitin ions for multiply-protonated states are discussed in this study.^15–17)^ The temperature dependence of the absolute reaction rate constant for proton transfer and the distribution of product ions were examined for those peptide and protein ions in the gas phase. An issue that is attracting considerable attention is their gas-phase conformations that could resemble structural evolution that originated from temperature in the gas phase. Based on experimental observations, we discuss the conformation changes in those ions with change in temperature. In addition, the relationship between gas-phase conformation, the self-solvation of protons by hydrophilic residues in polypeptide chains, and delocalization of charges with self-solvation are discussed.

## EXPERIMENTAL

Details of the experimental apparatus used in the present study have been described elsewhere.^15–17)^ Briefly, the system consists of four vacuum chambers with an ESI source, a tandem mass spectrometer, and a gas cell equipped with an octapole ion trap. Multiply-protonated peptide and protein ions were produced by ESI. A specific charge state of ions was selected by a quadrupole mass spectrometer (QMASS). The charge-selected ions emerging from QMASS were admitted to the gas cell, which was equipped with an octapole ion trap. The gas cell was filled with He along with the gaseous target molecules (TM). As TM, primary amines, secondary amines, and aromatic amines were used to collide with ions and to induce proton transfer reactions. Temperature dependence of the reaction rate constants and branching fractions for proton transfer from the charge-selected ions to TM was measured, by changing the temperature of the gas cell in the range of 280–460 K. Parent and product ions were extracted from the gas cell, and were mass-analyzed by a time-of-flight mass spectrometer equipped with a reflectron.

## RESULTS AND DISCUSSION

### Angiotension I

**Figure 1:**
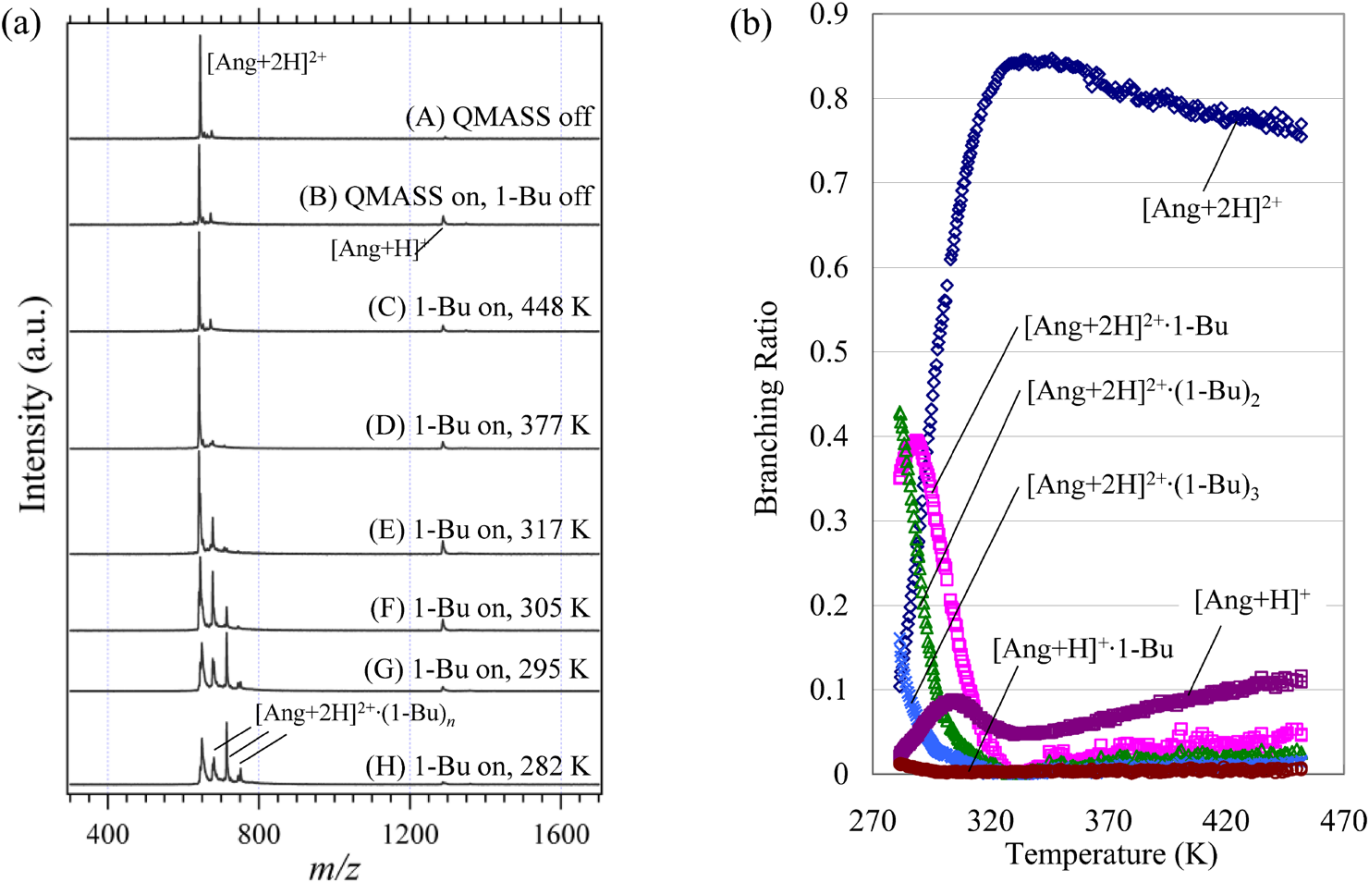
Fig. 1. (a) Time-of-flight mass spectra of [Ang+2H]^2+^ reacted with 1-Bu at various temperatures. (A) Ions of a specific charge state, [Ang+2H]^2+^, were selected with QMASS as a reactant. (B)–(H) Mass spectra of [Ang+2H]^2+^ reacted with 1-Bu at various temperatures. (b) In the reaction of [Ang+2H]^2+^ with 1-Bu, the branching ratios of parent and product ions are plotted as a function of temperature in the gas cell. (Reproduced with permission from ref 15.)

Proton transfer reactions of angiotensin I ions for the 2+ charge state, [Ang+2H]^2+^ were studied.^15)^ In Fig. 1 (a), time-of-flight mass spectra of [Ang+2H]^2+^ reacted with 1-butylamine (1-Bu) are shown as a function of mass-to-charge ratio (*m*/*z*). In (A) of Fig. 1 (a), an ion of specific charge states, [Ang+2H]^2+^, was selected with QMASS as a reactant. In (B)–(H), mass spectra of [Ang+2H]^2+^ reacted with 1-Bu at various temperatures are presented. The temperature of the gas cell was (B) 448, (C) 377, (D) 317, (E) 305, (F) 295, (G) 285, and (H) 282 K, respectively. As seen in the figures, a product ion, [Ang+H]^+^, was observed. Not shown in the figure, a protonated molecular ion, 1-Bu⋅H^+^, and a protonated dimer ion, (1-Bu)_2_⋅H^+^, were also detected at a lower mass range. It can therefore be assumed that a proton transfer reaction from [M+*z*H]*^z+^* to TM proceeds in the gas cell;
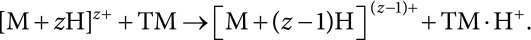
(1)

In Fig. 1 (b), branching ratios of parent and product ions are plotted as a function of temperature in the gas cell. The abundance of parent ion, [Ang+2H]^2+^, increased gradually with decreasing temperature from 470 to 330 K, and then decreased sharply at temperatures below 330 K. Complex ions, [Ang+2H]^2+^⋅(1-Bu)*_n_* (*n*=1–3), appeared at 330 K, and their abundances abruptly increased at temperatures below 330 K. The abundances of [Ang+H]^+^ gradually decreased with decreasing temperature from 470 to 330 K, and then increased rapidly from 330 to 300 K, whereas it abruptly decreased at temperatures below 300 K. The enhancement and suppression of proton transfer at 330 and 300 K appeared to be related to the appearance of [Ang+2H]^2^*^+^*⋅(1-Bu)*_n_.*

**Figure 2:**
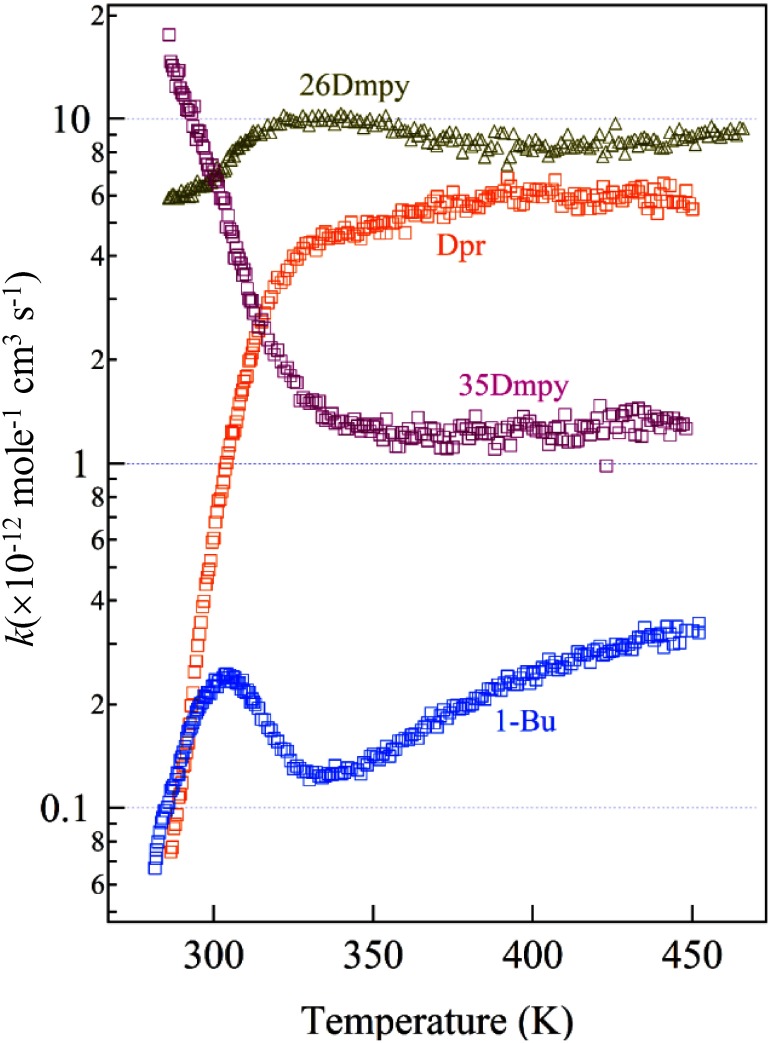
Fig. 2. Reaction rate constants of proton transfer from [Ang+2H]^2+^, to 1-Bu, Dpr, 35Dmpy, and 26Dmpy are plotted as a function of temperature in the gas cell. (Adapted with permission from ref 15.)

The absolute reaction rate constants, *k*, for proton transfer from [Ang+2H]^2+^ to gaseous TM were determined by Equation (1). Details of scheme used to determine absolute values of the rate constants have been described elsewhere.^16,17)^ In Fig. 2, the reaction rate constants for proton transfer from [Ang+2H]^2+^, to 1-Bu, dipropylamine (Dpr), 3,5-dimethylpyridine^30)^ (35Dmpy), and 2,6-dimethylpyridine (26Dmpy) are plotted as a function of temperature in the gas cell. The rate constant for [Ang+2H]^2^*^+^* that had reacted with 1-Bu gradually decreased with decreasing temperature from 470 to 330 K, then abruptly increased from 330 to 300 K, then dropped rapidly at temperatures below 300 K. The sudden increase in the rate constant below 330 K corresponds to complex formation, as shown in Fig. 1(b). Similar features were observed for the reactions with 1-pentylamine and *tert*-butylamine. The reaction appears to proceed *via* a two-step process with complex formation as a reaction intermediate. The enhancement in proton transfer is due to the favorable formation of this complex. The suppression of proton transfer is due to the stabilization of the complex. The rate constant for the reaction of [Ang+2H]^2^*^+^* with Dpr gradually decreased with decreasing temperature from 470 to 330 K, and then decreased sharply at temperatures below 330 K. Similar features were obtained for the reactions with diethylamine. Stabilization of the complex suppresses the reaction. The rate constant for the reaction of [Ang+2H]^2^*^+^* with 35Dmpy decreased slightly with decreasing temperature from 470 to 330 K, but then increased rapidly at temperatures below 330 K. Similar features were obtained for the reactions with pyridine, 2-methylpyridine. However, the rate constant for 26Dmpy slightly decreased with decreasing temperature from 470 to 360 K, increased slightly from 360 to 330 K, then decreased gradually at temperatures below 330 K, which was completely different from the values for 35Dmpy. When the temperature is decreased below 330 K, the structure of the peptide ions changes from elongated to compact conformations. There are two factors that could be at play in terms of enhancing or suppressing the proton transfer reaction, which are influenced by conformational changes related to temperature. One factor is steric hindrance. Protons that are attached to peptide ions with compact conformations would be located inside these sites. TM molecules are blocked to approaching protons, thus suppressing the reaction. Another factor is charge delocalization. Hydrophilic groups bind protons and the proton charge would be delocalized by those hydrogen bonds, so called self-solvation. Proton hopping, which occurs along hydrogen bonds with hydrophilic groups on a polypeptide chain, enhances the reactions. These two factors are closely related to the geometric structures and proton affinities (PA) of TM. In secondary amines such as Dpr, which contains two alkyl chains, steric hindrance is more effective than charge delocalization. Therefore, the reaction with Dpr is suppressed in the low temperature range. In protonated aromatic amine ions such as 35Dmpy, the charge is delocalized into the conjugated π electron system, which interacts with lone-pair electrons at a nitrogen atom. There is no steric hindrance by the two methyl groups on 35Dmpy, and suppression by steric hindrance in the peptide ion is less effective than enhancement by charge delocalization. Therefore, the reaction is enhanced in the low temperature range. On the other hand, in 26Dmpy, but not 35Dmpy, steric hindrance by the two methyl groups close to the nitrogen atom is possible and the effects of enhancement and suppression are compensated. In primary amines such as 1-Bu, there is one alkyl chain bound to the nitrogen atom, resulting in only minor steric hindrance. The PA of 1-Bu is relatively small, compared with those of Dpr, 35Dmpy, and 26Dmpy. Therefore, the reaction is enhanced by charge delocalization with decreasing temperature below 330 K, whereas it is suppressed by steric hindrance at temperatures below 300 K.

### Disulfide-intact and -reduced lysozyme

**Figure 3:**
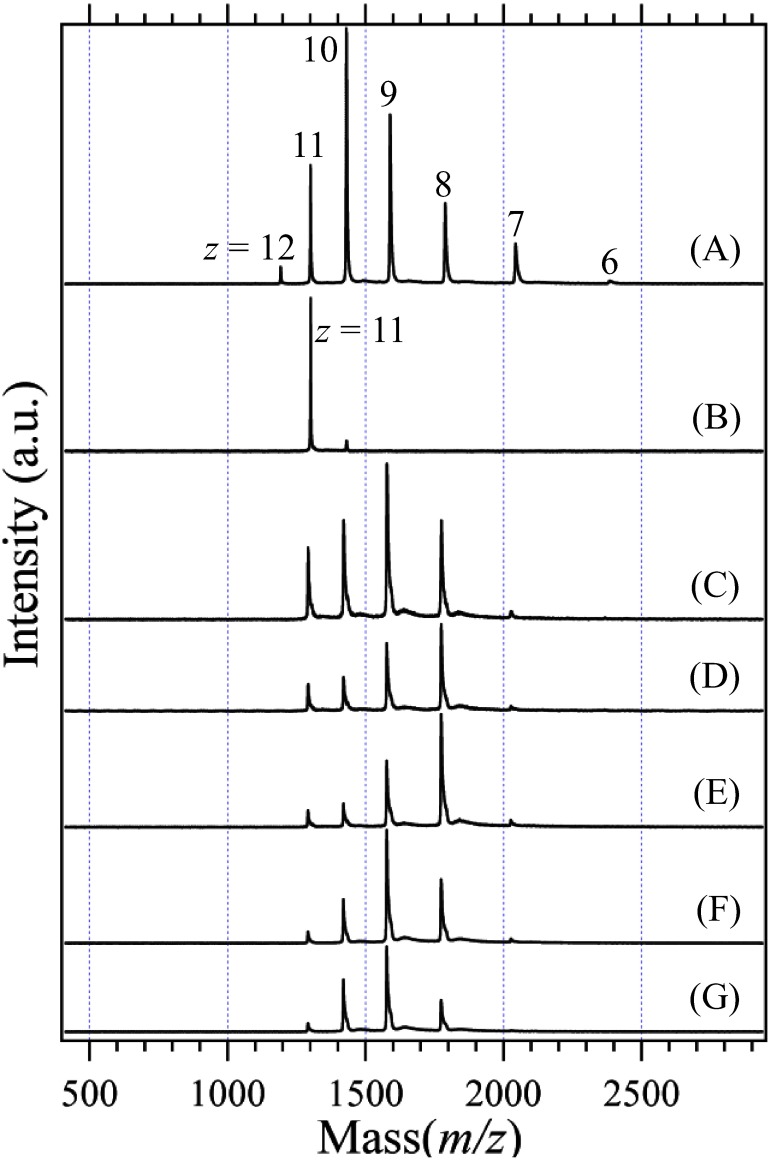
Fig. 3. Time-of-flight mass spectra of disulfide-intact lysozyme ions for an 11+ charge state, [Lys_i_+11H]^11+^ reacted with 26Dmpy at various temperature. (A) Mass spectrum of all ions produced with ESI, where DC voltage in QMASS was put off. (B) Ions of a specific charge state, [Lys_i_+11H]^11+^, were selected with QMASS as a reactant. (C)–(G) Mass spectra of [Lys_i_+11H]^11+^ reacted with 26Dmpy at various temperatures. Temperature of gas cell was (C) 460, (D) 410, (E) 360, (F) 310, and (G) 290 K, respectively. (Reproduced with permission from ref 16.)

**Figure 4:**
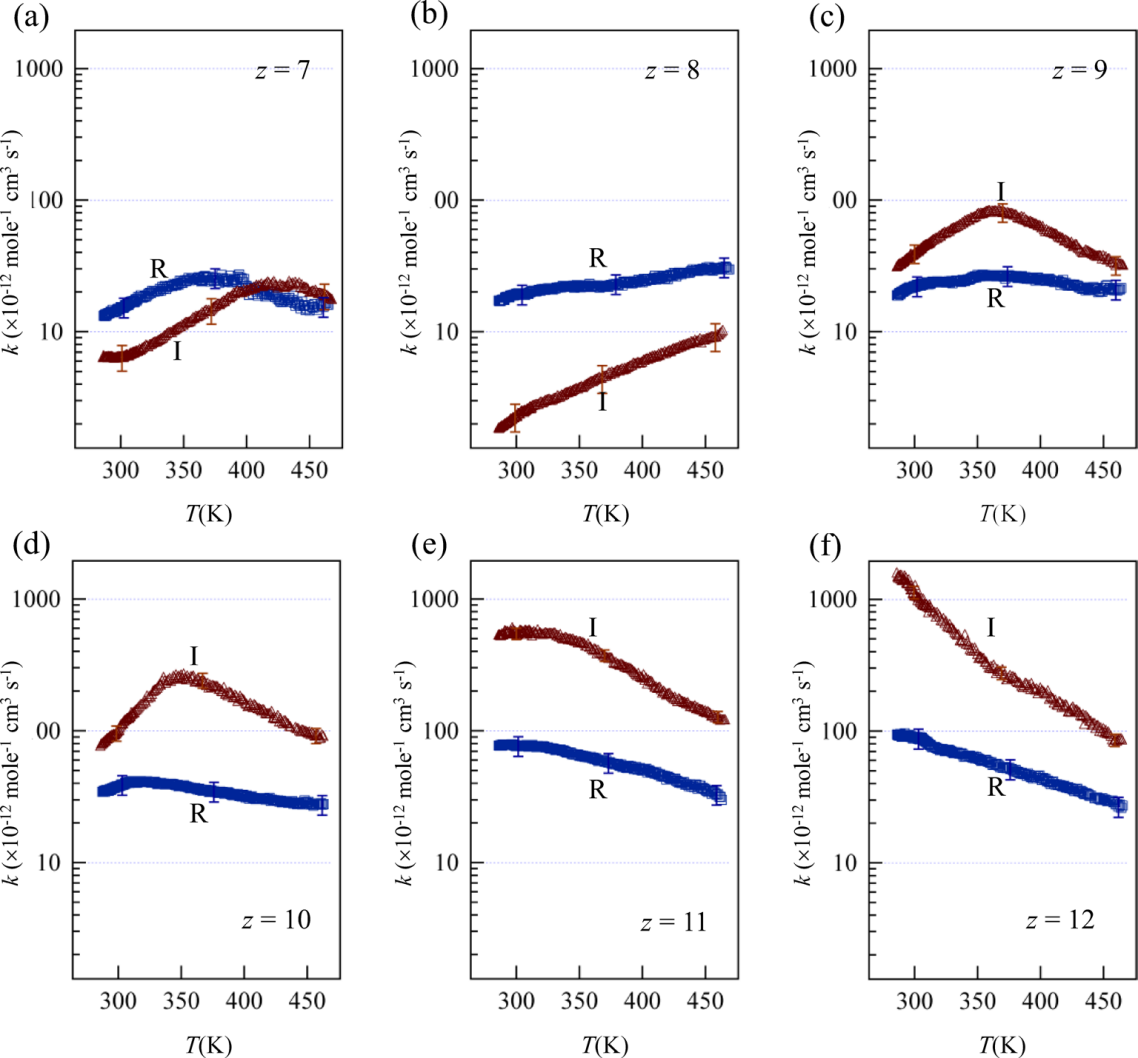
Fig. 4. The reaction rate constants for disulfide-intact and -reduced lysozyme ions, [Lys_i_+*z*H]*^z+^* and [Lys_r_+*z*H]*^z+^* respectively, are plotted as a function of temperature in the gas cell, for comparing the same charge states. The rate constants for 7+, 8+, 9+, 10+, 11+, and 12+ charge states are presented in (a), (b), (c), (d), (e), and (f), respectively. In the figures, letters represented “I” indicate the rate constants of [Lys_i_+*z*H]*^z+^*, whereas letters represented “R” indicate those of [Lys_r_+*z*H]*^z+^*. (Reproduced with permission from ref 16.)

Proton transfer reactions of disulfide-intact and -reduced lysozyme ions, [Lys_i_+*z*H]*^z^*^+^ and [Lys_r_+*z*H]*^z^*^+^, respectively, (7+ through 14+) to 26Dmpy were examined.^16)^ The disulfide-reduced form of lysozyme was obtained by boiling a lysozyme in an aqueous 0.01 M dithiothreitol (DTT) solution for 30 min.^16)^ Time-of-flight mass spectra of the disulfide-intact lysozyme ions for a 11+ charge state, [Lys_i_+11H]^11+^ reacted with 26Dmpy at various temperatures are shown in Fig. 3. A mass spectrum of multiply-protonated lysozyme ions, [Lys_i_+*z*H]*^z+^* (*z*=6–12), is shown in Fig. 3(A). In Fig. 3(B), ions in specific charge states, [Lys_i_+11H]^11+^, were selected as reactants with QMASS. Mass spectra of [Lys_i_+11H]^11+^ reacted with 26Dmpy at various temperatures, are shown in in Figs. 3(C)–(G). The target molecule, 26Dmpy was introduced into the gas cell along with He. The temperature of the gas cell was (C) 460, (D) 410, (E) 360, (F) 310, and (G) 290 K, respectively. As shown in the figures, drastic changes in the intensities of the product ions, [Lys_i_+*z*′H]*^z^*^′^*^+^* (*z*′=7–10), with decreasing temperature were observed. Not shown in these figures, a protonated molecular ion, 26Dmpy⋅H^+^, and a protonated dimer ion, (26Dmpy)_2_⋅H^+^, were also detected. In Fig. 4, the rate constants for [Lys_i_+*z*H]*^z+^* and [Lys_r_+*z*H]*^z+^* are plotted as a function of temperature, for comparing the same charge states. The rate constants for the 7+, 8+, 9+, 10+, 11+, and 12+ charge states are presented in Figs. 4(a), (b), (c), (d), (e), and (f), respectively. In the figures, letters represented as “I” indicate the rate constants of [Lys_i_+*z*H]*^z+^*, whereas letters represented as “R” indicate those of [Lys_r_+*z*H]*^z+^*. As shown in the figures, for the 9+, 10+, 11+, and 12+ charge states, the rate constants for [Lys_i_+*z*H]*^z+^* were larger than those of [Lys_r_+*z*H]*^z+^*. In the high temperature range, the rate constant of [Lys_i_+7H]^7+^ was equal to that of [Lys_r_+7H]^7+^, while it was smaller than that in the low temperature range. On the other hand, the rate constant of [Lys_i_+8H]^8+^ was much smaller than that of [Lys_r_+8H]^8+^. For 9+ and 10+ charge states, there were humps in the line shapes of [Lys_i_+*z*H]*^z+^*, whereas the line shapes of [Lys_r_+*z*H]*^z+^* were smoothly curved lines. As a whole, the rate constants for [Lys_i_+*z*H]*^z+^* changed more drastically than those for [Lys_i_+*z*H]*^z+^* with a change in temperature and for the charge state. For disulfide-intact ions, [Lys_i_+*z*H]*^z+^*, they increased more rapidly with increasing charge state compared to those for disulfide-reduced ions, [Lys_r_+*z*H]*^z+^*. It is noteworthy that the reaction rate constant of [Lys_i_+7H]^7+^ was much larger than that of [Lys_i_+8H]^8+^ over the entire temperature range. The rate constants for [Lys_i_+*z*H]*^z+^* changed more drastically with change in temperature than those for [Lys_r_+*z*H]*^z+^*. Drastic changes in the rate constants for [Lys_i_+*z*H]*^z+^* are related to restricted protein conformation by disulfide bonds.

### Ubiquitin

**Figure 5:**
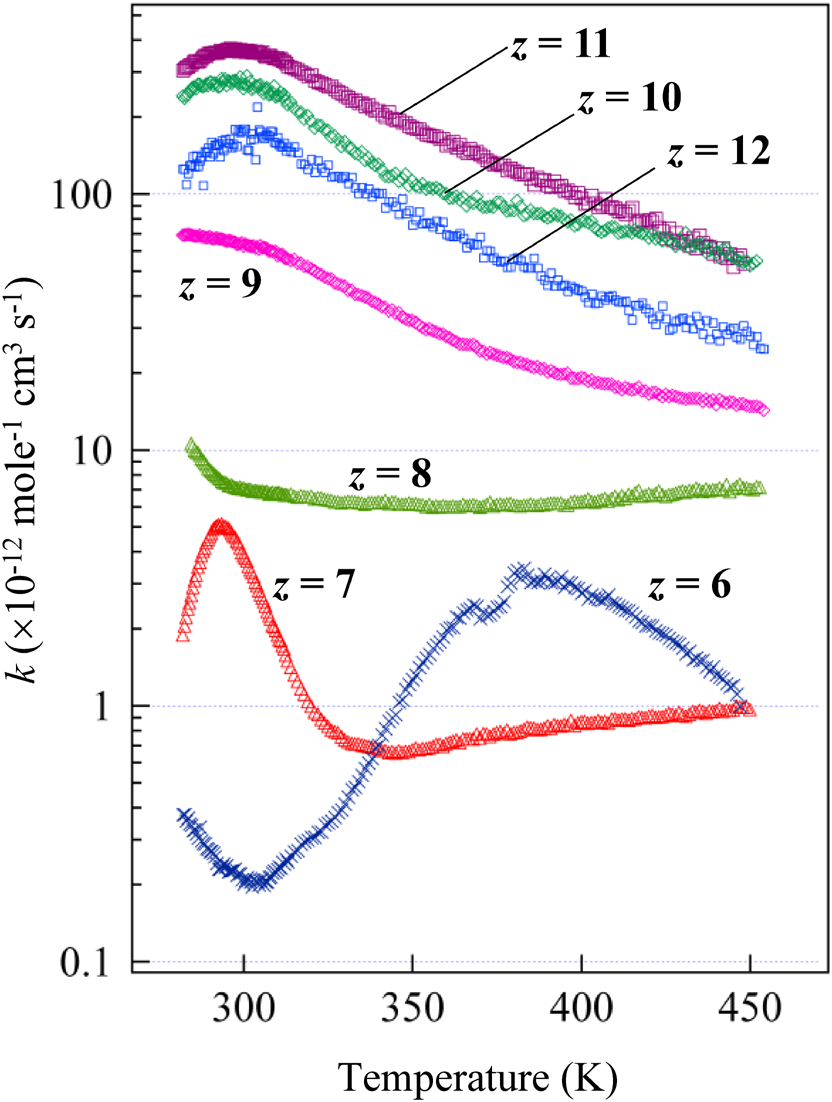
Fig. 5. Absolute reaction rate constants for proton transfer for [Ubi+*z*H]*^z^*^+^ (*z*=6–12) are plotted as a function of temperature in the gas cell. (Adapted with permission from ref 17.)

Proton transfer from multiply-protonated ubiquitin ions, [Ubi+*z*H]*^z^*^+^ (*z*=6–12), to 1-Bu was examined in the gas phase.^17)^ Absolute reaction rate constant for proton transfer was determined from the intensities of parent and product ions in the mass spectra. In Fig. 5, the reaction rate constants for [Ubi+*z*H] *^z^*^+^ (*z*=6–12), are plotted as a function of temperature in the gas cell. For multiply-protonated protein ions, the reaction rate constant for proton transfer generally grows larger with an increase in charge state, *z*. However, exceptionally, the reaction rate constant for [Ubi+6H]^6+^ was found to be much larger than that of [Ubi+7H]^7+^ in *T*=350–450 K. For [Ubi+6H]^6+^, it increased abruptly with decreasing temperature from 450 to 380 K, and also decreased from 370 to 300 K. Gas-phase conformations of ubiquitin ions have been determined by means of ion mobility measurements.^10)^ Those for lower charged states, *z*=6, 7, are compact in the low temperature range, whereas they adopt more unfolded and elongated conformations with increasing temperature. Remarkable changes in the reactivities of the ions with lower charge states are related to structural transitions from elongated to compact conformations that occur with decreasing temperature. The reaction is enhanced by charge delocalization and is suppressed by steric hindrance. A similar feature was observed in the case of the reaction of [Ang+2H]^2^^+^ with 1-Bu, which was described above.

## CONCLUSION

Proton transfer reactions of multiply-protonated angiotensin I, disulfide-intact and -reduced lysozyme, and ubiquitin ions to primary, secondary and aromatic amines were examined. By changing the temperature of the gas cell, it was possible to measure the temperature dependence of the absolute reaction rate constants for proton transfer. The reaction of [Ang+2H]^2+^ was enhanced and reduced by complex formation. The rate constants for [Lys_i_+*z*H]*^z^*^+^ changed more drastically with changes in the charge states and temperature than those for [Lys_r_+*z*H]*^z^*^+^, which can be attributed to the stabilization of the protein conformations by disulfide bonds. Drastic changes in the rate constants for [Ubi+*z*H]*^z^*^+^ in lower charge states are related to conformational transitions from elongated to compact conformations. For the ions with lower charge states in the lower temperature range, which have compact conformations, the two factors compete to enhance or to suppress the reaction. Delocalization of the proton charge enhances the reactions, whereas steric hindrance which prevents TM from accessing protons suppresses them.

## Notes

Mass Spectrom (Tokyo) 2020; 9(1): A0083
